# Current status and influencing factors of motor-cognitive risk syndrome in the older rural Chinese population: a cross-sectional study

**DOI:** 10.3389/fpubh.2025.1604019

**Published:** 2025-09-04

**Authors:** Jia Song, Teng Yang, Luhao Liu, Ping Ju, Xueting Wang, Lijuan Yang, Minmin Leng

**Affiliations:** ^1^Shandong Provincial Hospital Affiliated to Shandong First Medical University, Shandong, Jinan, China; ^2^School of Nursing, Shandong University of Traditional Chinese Medicine, Shandong, Jinan, China; ^3^School of Nursing, Shandong First Medical University, Shandong, Taian, China; ^4^Department of Nursing, Shandong Provincial Hospital Affiliated to Shandong First Medical University, Shandong, Jinan, China

**Keywords:** motor-cognitive risk syndrome, older adults, influencing factors, cross-sectional study, rural

## Abstract

**Objective:**

This study aims to screen for motor-cognitive risk syndrome (MCR) and analyze its influencing factors in rural older population in China, providing a reference for developing effective early intervention strategies.

**Methods:**

A total of 5,389 rural older adults from 33 villages in Xintai City, Shandong Province, China, were investigated using a convenience sampling method. We collected demographic information, subjective cognitive decline, gait speed, sleep quality, cognitive function, chronic pain, self-care ability, fear of falling, loneliness, nutritional status, depression, activities of daily living and social support. In this study, rural older adults were divided into an MCR group and a healthy control group. Chi-square tests, *t*-tests and rank sum tests were used to compare the differences in demographic characteristics between the two groups. Multivariate and linear logistic regression analyses was used to explore the factors influencing MCR in the rural older adults.

**Results:**

A total of 3,678 rural older adults were included in this study. The prevalence rate of MCR was 11.66%. The results revealed that chronic pain, age, falls, depression, social support, living conditions, medication types, vision loss, and chronic diseases were influencing factors of MCR in rural older population (*p* < 0.05).

**Conclusion:**

The prevalence rate of MCR in the rural older population is 11.66%, although its associated problems are more serious. Therefore, scientific interventions should be developed for rural older population to improve their motor and cognitive function, prevent dementia, and enhance their health quality of life.

## Background

Dementia is a clinical diagnosis defined by cognitive symptoms that interfere with the ability to carry out usual activities (1). Currently, approximately 50 million people worldwide are living with dementia. The number of dementia patients is expected to triple by 2050, with two-thirds coming from low-income and middle-income countries (LMICs) (2). Dementia is a major neurocognitive disorder characterized by decreased memory, problem-solving, language, and other cognitive skills. It can cause a series of complications (such as diabetes and cardiovascular diseases) and impose a heavy economic burden on society and families (2, 3).

The motoric cognitive risk syndrome (MCR) is the absence of dementia or motor impairment, and older adults experiencing subjective cognitive complaints and slow gait. Normal aging and mild cognitive impairment (MCI) constitute a new predictive syndrome of predementia. MCR can predict the occurrence of adverse health outcomes such as dementia, falls, disability and even death in older adults (4, 5). Therefore, as an “ultra-early” intervention window for preventing and treating dementia, effective measures should be taken to intervene before dementia. This can ensure that individuals remain in the preclinical stage and do not progress to dementia, thus effectively ensuring their quality of life (6). Epidemiological studies have shown that approximately 10% of older adults worldwide are affected by motor-cognitive syndrome. The overall prevalence ranges from 2 to 27%, with approximately 9% of older Asians being affected (6, 7). The risk of future dementia in patients with MCR is approximately three times greater than that in older adults without MCR (8), and the risk of death, falls, and disability is significantly increased (7). Therefore, MCR has gradually become a critical clinical and public health problem that threatens the health of older adults.

The risk factors for motor-cognitive risk syndrome are controllable, and behavioral changes can promote the improvement or even reversal of motor-cognitive risk syndrome. Currently, research on MCR in China is still in its infancy, with few large-scale epidemiological studies available. Epidemiological studies on MCR have been conducted primarily in Europe, the United States, and Japan. Affected by factors such as region, ethnicity, selection of research subjects, and sample size, the epidemiological data of MCR in the older adults differ across countries (9). Due to the non-equalization of basic public health services between urban and rural areas (10), health services in rural areas have problems of insufficient supply and low quality compared with urban areas. The older adults in rural areas usually have less health service resources and utilization rate than the older adults in urban areas, and it is more difficult to obtain timely and effective medical care. The Malaysian study (11) demonstrated that chronic conditions and reduced instrumental activities of daily living (IADL) may elevate the risk of MCR. Complementing these findings, the UK CFAS-Wales cohort identified a significant association between depressive or anxiety symptoms and increased MCR risk (12, 13). Furthermore, an American investigation (14) extended this evidence base by reporting that older adults with poor sleep quality exhibit a higher risk of MCR compared to those with good sleep quality. Notably, while MCR is also linked to adverse outcomes such as falls and malnutrition, the causal relationships between MCR and these sequelae remain insufficiently investigated (13). However, the prevalence and risk factors for MCR among rural older adults in China are not well-known. Therefore, in this study, MCR in older adults in rural areas of China was screened, and demographic, physiological, psychological, lifestyle, nutritional status, disease status, and medication history data were comprehensively analyzed to identify the factors influencing MCR in older adults and provide a reference for developing effective early intervention strategies.

## Methods

he participants were selected using the convenience sampling method, and the study was conducted among 5,389 rural older adults in 33 villages in Dongdu Town, Xintai City, Shandong Province, China, from April 2024 to June 2024. All rural older adults who met the inclusion criteria were screened in 33 villages. The inclusion criteria were as follows: ① aged ≥ 60 years; ② lived in the survey area for 1 year or more; ③ participated in local health check-ups and had health reports; ④ were able to communicate normally and cooperate to complete the survey items; and ⑤ were willing to participate in the survey. The exclusion criteria were as follows: ① MMSE score: junior high school and above ≤24 points, primary school ≤20 points, and illiterate ≤17 points (10); ②diagnosed with other neurodegenerative or neurological diseases; ③ participated in other interventional studies or drug trials; ④ had a history of mobility impairment or the use of a walking aid; ⑤ were repeated participants.

### Survey tools

#### General information questionnaire

The data included demographic data (age, sex, education level, monthly income, marital status, etc.), chronic disease conditions, polypharmacy, self-rated health status, lifestyle habits, BMI index, etc. According to the characteristics of the Asian population, a BMI ≥ 25 kg/m^2^ was defined as obesity in this study (5).

#### Pittsburgh Sleep Quality Index (PSQI)

The Pittsburgh Sleep Quality Index (PSQI) (15) is used to assess the sleep quality of older adults. The PSQI consists of seven dimensions, including subjective sleep quality, sleep latency, sleep duration, sleep efficiency, sleep disturbances, use of sleeping medication, and daytime dysfunction, with a total of 19 items. The scale evaluates the sleep status of the subjects over the past month, with a total score of 21 points, where a higher score indicates poorer sleep quality. Clinically, a score of 7 is generally used as the threshold for sleep quality, with ≤7 indicating good sleep quality and >7 indicating poor sleep quality.

#### Mini-Mental State Examination (MMSE)

The Mini-Mental State Examination (16) was used to evaluate the cognitive function of the older adults. The test included orientation, memory, attention and calculation ability, language ability, and visuospatial ability. The total score is 30 points; the higher the score, the better the cognitive function.

#### Visual Analogue Scale (VAS)

Pain intensity is represented by a score from 0 to 10. No pain at all is 0; mild tolerable pain is 1–3; moderate pain still tolerable is 4–6; and severe pain that is intolerable and affects sleep is 7–10.

#### Self-Care Ability Scale for the Elderly (SASE)

This study utilized the Chinese version of the Self-Care Ability Scale for the Elderly (17), which is suitable for all older adults. The scale includes assessments of daily activities, dressing, personal hygiene, shopping, safety, loneliness, physical strength, doing housework, and the environment, with three dimensions (skills, goals, and environment) and 17 items. It employs a 5-point Likert scale ranging from “strongly disagree” to “strongly agree,” corresponding to scores of 1–5, respectively. The total score ranges from 17 to 85, with higher scores indicating more self-care ability and a greater potential in older adults.

#### The Short FES-I (Short Falls Efficacy Scale-International)

This scale was developed by the European Group for Prevention of Falls in 2005 (18). Chinese scholars conducted reliability and validity tests of this scale on patients with cerebral infarction. The results showed that the scale had good reliability and validity (Cronbach’s α = 0.98) (19). It is a self-assessment scale consisting of two dimensions, namely, indoor and outdoor activities, with 16 items used to assess an individual’s fear of falling.

#### UCLA Loneliness Scale (UCLA-6)

This six-item scale was simplified by Guo et al. (19) and is based on the ULS-8. It is commonly used to assess loneliness in older adults and is suitable for community-dwelling older adults. Each item is scored on a 4-point Likert scale, with a total score ranging from 6 to 24. Higher scores indicate greater loneliness.

#### Short physical performance battery (SPPB)

The SPPB, developed by Rossi et al. (20), can evaluate three actual physical function measurements. This test includes a balance test, a sit-up test, and a walking speed test. Each test is scored from 0 (unable to complete) to 4 (best possible performance). The total SPPB score is 12, with a lower score indicating a greater likelihood of frailty.

#### Short-Form Mini Nutritional Assessment (MNA-SF)

The Short-Form Mini Nutritional Assessment (21), which includes six questions on dietary and weight changes, mobility, stress or acute illness, mental status, and body mass index, is used for evaluation. The total score is 14 points, and a score of ≤11 indicates a decline in vitality.

#### Patient Health Questionnaire-9 (PHQ-9)

The Patient Health Questionnaire-9 (PHQ-9) consists of 9 items (22), including loss of interest in pleasurable activities, feeling depressed, sleep disturbances, lack of energy, eating disorders, low self-esteem, difficulty concentrating, slow movement, and negative thoughts. It assesses the subject’s feelings over the past 2 weeks. Each item is scored from 0 to 3, with a total score ranging from 0 to 27. The higher the score is, the more severe the depressive symptoms.

#### The Activity of Daily Living Scale (ADL)

The Activity of Daily Living Scale (ADL) (23) is used to assess the daily living abilities of older adults. ADL consists of two parts: the physical activities of daily living (6 domains) and the instrumental activities of daily living (8 domains), totaling 20 items. Each item is scored on a four-point scale ranging from “can do independently” to “completely unable to do,” ranging from 20 to 80 points. A lower score indicates better daily living ability, and clinically, an ADL total score of ≤26 is considered to indicate normal daily living ability.

#### Social Support Rating Scale (SSRS)

The Social Support Rating Scale (24) is used to assess the level of social support among older adults. The SSRS consists of 10 items divided into three dimensions: subjective support, objective support, and the degree of support utilized. The total score ranges from 12 to 66 points, with 12–22 points indicating a low level of social support, 23–44 points indicating a moderate level, and 45–66 points indicating a high level of social support. The higher the score is, the better the level of social support.

#### Clinical Dementia Rating (CDR)

The Clinical Dementia Rating (25) is a standardized instrument used to assess cognitive function and functional abilities in dementia patients. Dementia severity is assigned based on a global score derived from six domains: memory, orientation, judgment and problem-solving, community affairs, home and hobbies, and personal care. The CDR score has 5 grades: no dementia is 0; suspicious dementia is 0.5; mild dementia is 1; moderate dementia is 2; severe dementia is 3.

### The definition of MCR

The MCR was evaluated according to the criteria proposed in the literature (5, 26). MCR was identified if the following four criteria were met: (1) Subjective cognitive complaints (SCC): In this study, “Have you experienced a decline in your memory lately?” was used to assess SCC. Those who answered “Yes” were judged to have SCC. (2) Slow walking speed: The diagnostic criterion for sarcopenia recommended by the European Working Group on Sarcopenia in the older adults (EWGSOP2) (27) was used in this study. Gait speed was assessed using the 4-m gait speed assay. A total of 2 measurements were performed, and the average value was taken. Gait speed ≤0.8 m/s was defined as decreased gait speed. (The study population exhibited comparatively reduced mean gait speed. Since one standard deviation (SD) below this mean corresponded to approximately 0.8 m/s, this threshold was selected as the slow gait criterion based on the empirical distribution). (3) There was no movement disorder, and the ability to perform activities of daily living was preserved. The ADL score was less than 22. (4) Free of dementia: absence of dementia diagnosis in physical examination reports (where available, these reports took diagnostic precedence); Clinical Dementia Rating (CDR) score of 0 or 0.5; Mini-Mental State Examination (MMSE) scores exceeding established education-adjusted thresholds: >24 for junior high school education or above, >20 for primary school education, and >17 for illiterate individuals.

### Data collection

Data collection was conducted by multiple researchers who had undergone uniform training. They introduced the purpose of the study to the older population using a unified set of instructions in a one-on-one survey format. After informed consent was obtained from the older population, the survey was carried out, and a unified method and equipment were used to measure the relevant indicators. After the survey, the questionnaires were collected on the spot and checked for accuracy before being archived. Questionnaires with incomplete or inconsistent information were considered invalid. The study subjects general information and laboratory indicators were collected by reviewing electronic health examination reports.

### Statistical methods

SPSS 26.0 statistical software was used for data processing. Participants were divided into 2 groups according to the MCR criteria. MCR group and healthy control group. Normally distributed measurement data are expressed as $\bar{x}$ ± s. *T*-test was used for comparisons between groups. Count data are expressed as relative numbers. The chi-square test was used for comparisons between groups. The factors influencing MCR were analyzed using linear logistic regression and multivariate logistic regression analyses. *p*-value of <0.05 was considered to indicate statistical significance.

## Results

### Screening process for research subjects

**Figure 1:**
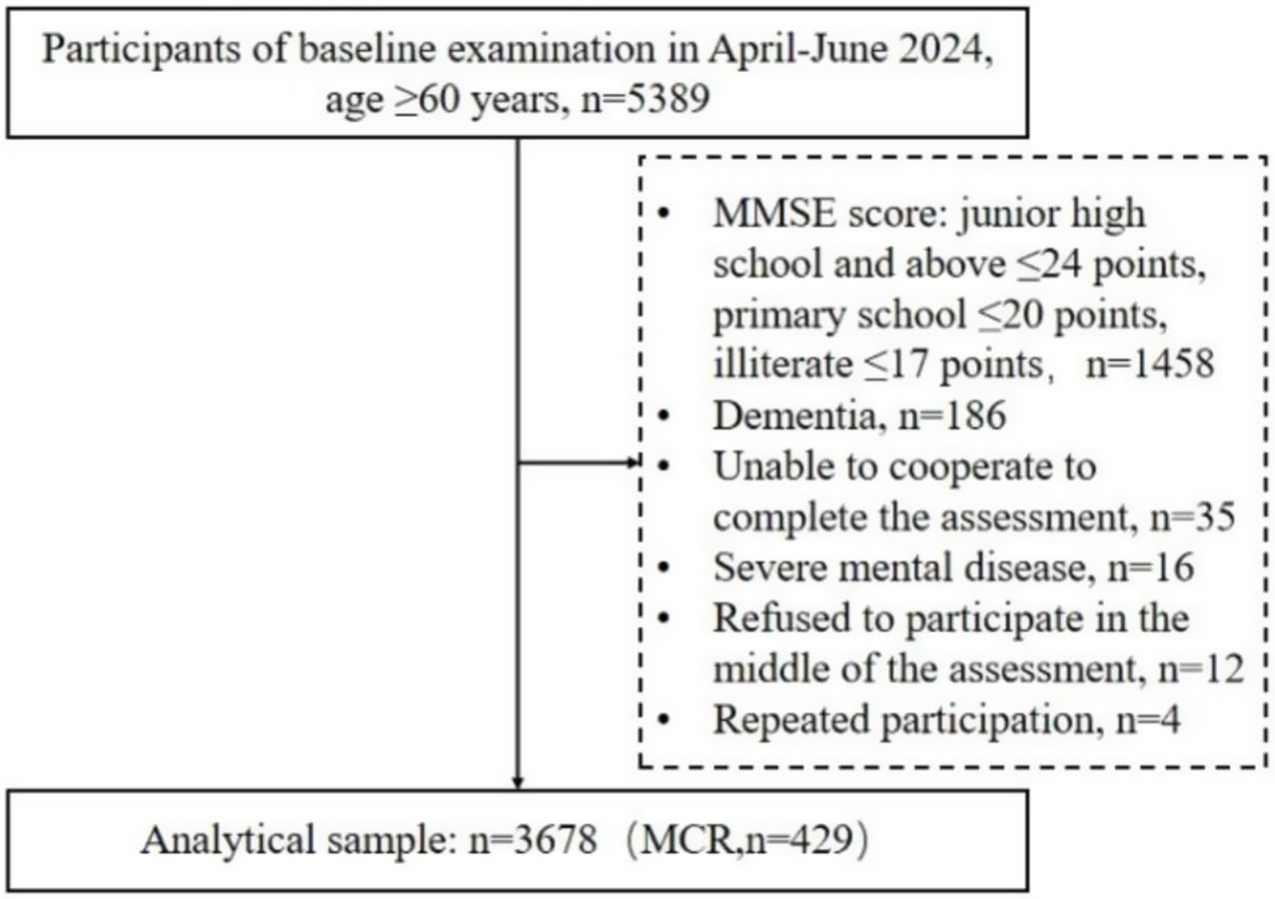
Flowchart of study participants.

This study conducted a questionnaire survey among 5,389 rural older adults. Ultimately, 3,678 rural older adults who met the inclusion criteria were included. Of these, 429 rural older adults were diagnosed with MCR. The screening procedure is detailed in Figure 1.

### Basic characteristics of the study participants

A total of 3,678 rural older adults were included. The ages ranged from 60 to 90 years, with an average age of 69.4 ± 6.12 years. In terms of sex, 1,535 (41.7%) were males, and 2,143 (58.2%) were females. Most participants were married (81%) and lived with their spouses (78%). The number of children was 2 (53.6%) or 3 or more (36.9%). In terms of education level, most of them were illiterate (45.6%) and had a primary school education (28.7%). The proportion of those with a monthly income < 1,000 yuan (70.6%) was significant. There were 429 rural older adults with MCR, and the prevalence rate was 11.66% (see Table 1 for details).

**Table 1:** Basic characteristics of MCR prevalence in the rural older population (*N* = 3,678).

Basic features	Number of respondents (*N* = 3,678)	MCR (*N* = 429)	Healthy control group (*N* = 3,249)	χ^2^	*P*
Sex				2.792	0.095
Male	1,535 (41.7%)	163 (38%)	1,372 (42.2%)		
Female	2,143 (58.3%)	266 (62%)	1877 (57.8%)		
Age				2518.791	<0.001
60–69	1932 (52.5%)	152 (35.4%)	1780 (54.8%)		
70–79	1,530 (41.6%)	226 (52.7%)	1,304 (40.1%)		
≥80	216 (5.9%)	51 (11.9%)	165 (5.1%)		
Marital status				26.019	<0.001
Married	2,981 (81%)	308 (71.8%)	2,673 (82.3%)		
Divorced	33 (0.9%)	6 (1.4%)	27 (0.8%)		
Widowed	654 (17.8%)	113 (26.3%)	541 (16.7%)		
Unmarried	10 (0.3%)	2 (0.5%)	8 (0.2%)		
Number of children				405.129	<0.001
None	15 (0.4%)	2 (0.5%)	13 (0.4%)		
1	329 (8.9%)	24 (5.6%)	305 (9.4%)		
2	1975 (53.7%)	194 (45.2%)	1781 (54.8%)		
≥3	1,359 (36.9%)	209 (48.7%)	1,150 (35.4%)		
Living arrangements				1847.322	<0.001
Live alone	648 (17.6%)	116 (27.0%)	532 (16.4%)		
Live with children	159 (4.3%)	22 (5.1%)	137 (4.2%)		
Live with a spouse	2,871 (78.1%)	291 (67.8%)	2,580 (79.4%)		
Educational level				228.905	<0.001
Illiterate	1,678 (45.6%)	223 (52.0%)	1,455 (44.8%)		
Primary school	1,057 (28.7%)	131 (30.5%)	926 (28.5%)		
Junior high school	602 (16.4%)	51 (11.9%)	551 (17.0%)		
High school/Technical Secondary school	312 (8.5%)	23 (5.4%)	289 (8.9%)		
Associate degree/University	29 (0.8%)	1 (0.2%)	28 (0.9%)		
Monthly income				1530.21	<0.001
<1,000	2,599 (70.7%)	341 (79.5%)	2,258 (69.5%)		
1,000–1999	434 (11.8%)	31 (7.2%)	403 (12.4%)		
2000–3,499	269 (7.3%)	26 (6.1%)	243 (7.5%)		
3,500–4,999	174 (4.7%)	14 (3.3%)	160 (4.9%)		
≥5,000	202 (5.5%)	17 (4.0%)	185 (5.7%)		
Do you drink?				1482.379	<0.001
Often	526 (14.3%)	49 (11.4%)	477 (14.7%)		
Occasionally	442 (12.0%)	46 (10.7%)	396 (12.2%)		
Used to drink but not anymore	464 (12.6%)	68 (15.9%)	396 (12.2%)		
Never	2,246 (61.1%)	266 (62.0%)	1980 (60.9%)		
Types of medication				64.833	<0.001
None	1,528 (41.5%)	141 (32.9%)	1,387 (42.7%)		
1	955 (26.0%)	103 (24.0%)	852 (26.2%)		
2	591 (16.1%)	78 (18.2%)	513 (15.8%)		
3	295 (8.0%)	40 (9.3%)	255 (7.8%)		
4	132 (3.6%)	25 (5.8%)	107 (3.3%)		
≥5	177 (4.8%)	42 (9.8%)	135 (4.2%)		
Do you have hearing loss?				24.569	<0.001
None	2,360 (64.2%)	229 (53.4%)	2,131 (65.6%)		
Yes	1,318 (35.8%)	200 (46.6%)	1,118 (34.4%)		
Is there a decrease in vision?				22.481	<0.001
None	1836 (49.9%)	168 (39.2%)	1,668 (51.3%)		
Yes	1842 (50.1%)	261 (60.8%)	1,581 (48.7%)		
Have you often experienced urinary incontinence in the past month?				2158.718	<0.001
Never	3,116 (84.7%)	329 (76.7%)	2,787 (85.8%)		
Occasionally (<1 time/week)	290 (7.9%)	45 (10.5%)	245 (7.5%)		
Regularly (2–3 times/week)	164 (4.5%)	33 (7.7%)	131 (4.0%)		
Consistently (≥1 time/day)	108 (2.9%)	22 (5.1%)	86 (2.6%)		
Do you usually suffer from constipation?				1728.092	<0.001
None	3,025 (82.2%)	326 (76.0%)	2,699 (83.1%)		
Mild	546 (14.8%)	77 (17.9%)	469 (14.4%)		
Moderate to severe	107 (2.9%)	26 (6.1%)	81 (2.5%)		
What is your health status?				2853.797	<0.001
Very good	1,054 (28.7%)	67 (15.6%)	987 (30.4%)		
Better	1,476 (40.1%)	180 (42.0%)	1,296 (39.9%)		
General	949 (25.8%)	132 (30.8%)	817 (25.1%)		
Not very good	176 (4.8%)	46 (10.7%)	130 (4.0%)		
Very bad	23 (0.6%)	4 (0.9%)	19 (0.6%)		
Do you do farm work?				2206.156	<0.001
Yes	2,435 (66.2%)	236 (55.0%)	2,199 (67.7%)		
No	990 (26.9%)	169 (39.4%)	821 (25.3%)		
Never	253 (6.9%)	24 (5.6%)	229 (7.0%)		
Obesity				1.699	0.192
Yes	1786 (48.6%)	221 (51.5%)	1,565 (48.2%)		
No	1892 (51.4%)	208 (48.5%)	1,684 (51.8%)		
Chronic diseases				22.850	<0.001
No	368 (10.0%)	15 (3.5%)	353 (10.9%)		
Yes	3,310 (90.0%)	414 (96.5%)	2,896 (89.1%)		
Food preferences				995.125	<0.001
Meat	327 (8.8%)	36 (8.3%)	291 (8.9%)		
Vegetarian food	1,539 (41.8%)	197 (45.9%)	1,342 (41.3%)		
Meat and vegetable pairing	1812 (49.2%)	196 (45.6%)	1,616 (49.7%)		

Chi-square values represent the comparisons between healthy controls and MCR patients; *P*-value of χ^2^ value < 0.05.

### Single factor analysis

The clinical data of the healthy control and the MCR groups were compared. There was no significant difference in the prevalence of MCR in rural older population based on sex, obesity status, sitting time or SASE score (*p* > 0.05). Other characteristics significantly affected the prevalence of MCR in rural older population (*p* < 0.05) (see Tables 1, 2 for details).

**Table 2:** Comparison of clinical data between healthy controls and patients with MCR.

Basic feature	Healthy control group (3249) *M* (P25, P75)^a^ *M* ± SD^b^	MCR (429) (95%CI)^a^ M ± SD^b^	*Z*/*t*	*P*
Sitting time^a^	2 (1,4)	(0,60)	0.432	0.665
VAS^a^	1.917 (0,3)	(0,10)	7.483	<0.001
PSQI^a^	6.92 (3,10)	(1,21)	4.759	<0.001
The Short FES-I^a^	12.23 (7,17)	(7,28)	6.462	<0.001
UCLA-6^a^	6 (6,6)	(6,24)	4.092	<0.001
PHQ-9^a^	2.20 (0,3)	(0,27)	3.742	<0.001
SASE^b^	59.61 ± 5.288	59.48 ± 5.440	0.481	0.631
SSRS^b^	43.03 ± 7.033	40.45 ± 7.366	7.118	<0.001
ADL^a^	20 (20,20)	(20,74)	4.315	<0.001
MMSE^a^	26 (22,28)	(18,30)	19.592	<0.001

MCR, motor cognitive risk syndrome; ^a^rank sum test; ^b^*t*-test; *M* (P25, P75), (95%CI) and *Z* value from rank sum test; *t*-value and *M* ± SD from *t*-test; M ± SD, mean ± standard deviation; *P*-value of *Z*/*t* value < 0.05; SSRS, Social Support Rating Scale; The Short FES-I, Short Falls Efficacy Scale-International; UCLA-6, UCLA Loneliness Scale; PHQ-9, Patient Health Questionnaire-9; SASE, Self-Care Ability Scale for the Elderly; VAS, Visual Analogue Scale; PSQI, Pittsburgh Sleep Quality Index; ADL, Activity of Daily Living Scale; MMSE, Mini-Mental State Examination.

### Multiple-factor analysis

Multivariate and linear logistic regression were used to analyze the influencing factors of MCR among the rural older population, with the occurrence of MCR as the dependent variable (assignment: occurrence = 1, non-occurrence = 2). The variables with statistically significant differences in Tables 1, 2 were used as independent variables to conduct linear and multivariate logistic regression analyses. The assignments for the multifactor analysis variables are detailed in Table 3. The results of the analysis are detailed in Table 4.

**Table 3:** Assignment table for the multifactor analysis variables.

Independent variable	Assignment method
Age	60 ~ 69 = 1; 70 ~ 79 = 2; ≥80 = 3
Sex	Male = 1; Female = 2
Marital status	Married = 1; Divorced = 2; Widowed = 3; Unmarried = 4
Number of children	None = 1; 1 = 2; 2 = 3; ≥3 = 4
Living arrangement	Live alone = 1; Children living together = 2; Live with a spouse = 3
Educational level	Illiterate = 1; Primary school = 2; Junior high school = 3; High school/Technical and vocational secondary school = 4; Associate degree/University = 5
Monthly income	<1,000 = 1; 1,000–1999 = 2; 2000–3,499 = 3; 3,500–4,999 = 4; ≥5,000 = 5
Do you drink?	Often = 1; Occasionally = 2; Used to drink but not anymore = 3; Never = 4
Types of medication	None = 1; 1 = 2; 2 = 3; 3 = 4;4 = 5; ≥5 = 6
Do you have hearing/vision loss?	None = 1; Yes = 2
Have you often experienced urinary incontinence in the past month?	Never Occasionally (<1 time/week) Regularly (2–3 times/week) Consistently (≥1 time/day)
Do you usually suffer from constipation?	None = 1; Mild = 2; Moderate to severe = 3
What is your health status?	Very good = 1; Better = 2; General = 3; Not very good = 4; Very bad = 5
Do you do farm work?	Yes = 1; No = 2; Never = 3
What are your dietary preferences?	Preference for meat dishes = 1; Vegetarian preference = 2; Combination of meat and vegetables = 3
Have you fallen in the past year?	No = 1; Yes = 2
Are you worried about falling?	No = 1; Mild = 2; A bit = 3; Very = 4

**Table 4:** Logistic regression analysis of influencing factors of MCR in the rural older population.

Independent variable	*B*	Standard error (B)	*t*/OR (95%CI)/Chi-square	*P*
VAS^a^	−0.011	0.002	−4.826	<0.001
PSQI^a^	0.000	0.001	−0.285	0.776
The Short FES-I^a^	−0.003	0.001	−2.748	0.006
UCLA-6^a^	−0.002	0.002	−0.728	0.467
PHQ-9^a^	0.005	0.002	2.368	0.018
SSRS^a^	0.003	0.001	3.175	0.002
Age				
60 ~ 69				
70 ~ 79	−0.752	0.214	0.471 (0.310–0.716)	<0.001
≥80	−0.370	0.191	0.691 (0.475–1.004)	0.053
Marital status				
Married				
Divorced	−0.972	1.013	0.378 (0.052–2.753)	0.337
Widowed	−0.604	1.103	0.547 (0.063–4.750)	0.584
Unmarried	−1.335	1.019	0.263 (0.036–1.940)	0.190
Number of children				
None				
1	−0.936	0.972	0.392 (0.058–2.637)	0.336
2	−0.358	0.242	0.699 (0.436–1.123)	0.139
≥3	−0.196	0.120	0.822 (0.650–1.040)	0.103
Living arrangements				
Live alone				
Live with children	0.656	0.253	1.926 (1.173–3.164)	0.010
Live with a spouse	0.543	0.296	1.722 (0.965–3.073)	0.066
Educational level				
Illiterate				
Primary school	0.720	1.044	2.055 (0.265–15.903)	0.490
Junior high school	0.724	1.043	2.063 (0.267–15.925)	0.487
High school/Technical secondary school	0.601	1.046	1.823 (0.235–14.174)	0.566
Associate degree/University	0.585	1.056	1.796 (0.227–14.220)	0.579
Monthly income				
<1,000				
1,000–1999	0.440	0.293	1.553 (0.875–2.757)	0.133
2000–3,499	0.120	0.340	1.127 (0.579–2.195)	0.724
3,500–4,999	0.329	0.342	1.390 (0.710–2.720)	0.336
≥5,000	0.127	0.391	1.135 (0.528–2.441)	0.746
Do you drink?				
Often				
Occasionally	−0.093	0.177	0.911 (0.644–1.288)	0.598
Used to drink but not anymore	−0.103	0.180	0.902 (0.635–1.283)	0.568
Never	0.188	0.163	1.207 (0.878–1.659)	0.248
Types of medication				
None				
1	−0.471	0.218	0.624 (0.407–0.958)	0.031
2	−0.545	0.220	0.580 (0.377–0.892)	0.013
3	−0.448	0.227	0.639 (0.410–0.997)	0.048
4	−0.572	0.256	0.564 (0.342–0.932)	0.025
≥5	−0.240	0.296	0.787 (0.441–1.405)	0.417
Do you have hearing loss?				
No				
Yes	−0.135	0.115	0.874 (0.698–1.095)	0.242
Is there a decrease in vision?				
No				
Yes	−0.248	0.115	0.780 (0.623–0.977)	0.030
Have you often experienced urinary incontinence in the past month?				
Never				
Occasionally (<1 time/week)	−0.356	0.264	0.700 (0.418–1.174)	0.177
Regularly (2–3 times/week)	−0.089	0.305	0.914 (0.503–1.662)	0.769
Consistently (≥1 time/day)	0.047	0.324	1.048 (0.555–1.980)	0.884
Do you usually suffer from constipation?				
None				
Mild	−0.475	0.248	0.622 (0.383–1.010)	0.055
Moderate to severe	−0.497	0.269	0.608 (0.359–1.031)	0.065
What is your health status?				
Very good				
Better	−0.317	0.615	0.729 (0.218–2.430)	0.606
General	0.181	0.602	1.199 (0.368–3.903)	0.763
Not very good	0.161	0.602	1.175 (0.361–3.826)	0.789
Very bad	0.650	0.618	1.915 (0.570–6.433)	0.293
Do you do farm work?				
Yes				
No	−0.075	0.255	0.928 (0.563–1.530)	0.770
Never	0.209	0.259	1.233 (0.742–2.048)	0.419
Chronic diseases				
No				
Yes	−0.708	0.284	0.493 (0.283–0.859)	0.013
Food preferences				
Meat				
Vegetarian food	0.020	0.192	1.020 (0.700–1.487)	0.918
Meat and vegetable pairing	0.191	0.107	1.210 (0.981–1.494)	0.076
HL Test^b^			7.857	0.448

^a^Linear logistics regression; the others are multivariate logistics regression analyses; *t* value from linear logistics regression; OR (95%CI) from multivariate logistics regression; 95% CI = 95% confidence interval; OR = odds ratio; *B* value represents the strength and direction of the statistical association between the independent variable and the dependent variable; Standard Error (B) indicates the precision of the regression coefficient estimate; *P*-value < 0.05; SSRS=Social Support Rating Scale; The Short FES-I=Short Falls Efficacy Scale-International; UCLA-6 = UCLA Loneliness Scale; PHQ-9 = Patient Health Questionnaire-9; VAS=Visual Analogue Scale; PSQI=Pittsburgh Sleep Quality Index; ^b^HL Test is Hosmer and Lemeshow Test (*P* > 0.05 provides no evidence against the null hypothesis of good fit); Chi-square from HL Test.

## Discussion

This study revealed that the prevalence rate of MCR in Chinese rural older population was 11.66%. The logistic regression results of this study showed that the risk factors for MCR in rural older population were advanced age, chronic pain, polypharmacy, depression, insufficient social support, living with children, decreased vision, chronic disease, and fear of falling (*p* < 0.05). The top three variables most strongly associated with MCR are age (70 ~ 79), chronic diseases (Yes), Living arrangements (living with children). The model had a good fit according to the Hosmer–Lemeshow test (*p* > 0.05).

### Prevalence of MCR in the rural older population

In this study, 3,678 rural older population were analyzed, with 429 having MCR. The prevalence rate was 11.66%. Compared with other regions within China, the prevalence of MCR was higher than in Beijing (9.6%) and western China (10.7%) but lower than in eastern China (12.7%) (28). When compared with international studies, the prevalence rates in developing countries (3.5–15%) are higher than those reported in the United States (5.7–10.35%) (28, 29) and European countries (2.56–9.92%) (30). Compared to Germany (25.3%) (31) and Mexico (14.74%) (23), the detection rates in this survey were low. The lowest incidence is in Malaysia (3.5%) (16), and the highest is in India (15%) (32). This may be attributed to variations in sampling methods, study types, assessment and grading techniques, sample size, selection of survey instruments, definitions of study subjects, and the economic, educational, cultural, and demographic characteristics across different countries.

### Influencing factors of MCR in the rural older population

In the present study, older population aged 70–79 were more likely to suffer from MCR. However, the prevalence of MCR did not increase with age. The results of a longitudinal study on health and aging in Mexico (48) revealed that the incidence of MCR increased exponentially with age. This finding is not consistent with the results of this study. Previous studies (15, 33) have shown that advanced age is a critical risk factor for MCR. Age acts as a catalyst in the pathogenesis of dementia through the MCR pathway. This may be because the relationship between biological aging and MCR stems from a common biological mechanism (33). Increasing age, loss of sensory and motoric function, neurological and lifestyle changes, and reduced gait speed and cognitive ability contribute to the increased risk of MCR in older adults (16). Nevertheless, inconsistencies persist across studies regarding the relationship between age and Motoric Cognitive Risk (MCR) incidence, suggesting this association merits further investigation. Notably, the present study included a limited cohort of octogenarians (aged ≥80 years), which may constrain the generalizability of our findings to this high-risk demographic and partially account for the observed heterogeneity in age-related correlations.

Older population with 1–4 types of medication were more likely to suffer from MCR. A large-sample cross-sectional study in the American community (34) reported that the incidence of MCR was 10% in people with polypharmacy (5 or more types of medication), and the incidence of MCR was higher in people with polypharmacy. However, this finding is not consistent with the results of this study. Studies have shown that polypharmacy is a risk factor for MCR and is associated with poorer physical and cognitive function in older adults (24). This may be due to the adverse health outcomes of brain metabolism, brain structure, gait and cognition caused by the use of multiple drugs in older population (34). However, whether more kinds of medicines used results in a higher likelihood of MCR remains to be further studied.

Older adults with chronic diseases are more likely to suffer from MCR. Previous studies (17) have shown that some chronic diseases (such as diabetes, stroke, heart disease, chronic obstructive pulmonary disease, and coronary artery disease) are associated with an increased risk of MCR in older adults, which is consistent with the results of this study. The pathological basis of MCR is related to white matter hyperintensities, frontal lacunar infarcts, and gray matter atrophy in the premotor and prefrontal cortex. These pathologies are related to chronic diseases, especially cardiovascular diseases. A variety of chronic diseases can exacerbate the pathological state (22).

Additionally, the inflammatory response is the pathological basis of MCR (22). Inflammatory markers adversely affect walking speed and cognitive impairment in older adults (18, 19, 35). However, older adults with chronic diseases are more likely to have a disorder of proinflammatory cytokines and are more likely to suffer from MCR. In addition, heart-related diseases can lead to regional cerebral hypoperfusion in the cognitive regulatory regions of the brain, resulting in destructive effects on cognition (20).

Older adults with depression are more likely to suffer from MCR. Some studies have shown that (11) depression is a risk factor for MCR, which is consistent with the results of this study. Studies have shown that social activity positively correlates with gait speed and physical function (36, 37). Older adults with depression may reduce their social activity level, which increases the risk of MCR. Additionally, the decrease in social networks caused by cognitive decline and slow gait speed in older patients with MCR has adverse effects on their mental health (38). Furthermore, the main symptom of MCR is SCC, which overlaps with symptoms related to depression (23). Therefore, depression may be closely related to the MCR diagnosis.

Older adults with insufficient social support are more likely to suffer from MCR. Studies have shown that (21) adequate social support can reduce the risk of MCR, which is consistent with the results of this study. This may be because tangible social support increases gray matter volume in brain regions and reinforces MCR-associated neural substrates, thereby reducing the risk of MCR (39). Older adults with vision loss are more likely to suffer from MCR. Studies have shown that a lack of physical activity affects the normalization of blood glucose levels and memory function (22). An older population with vision loss may reduce physical activity to avoid injury, which affects cognitive function.

Older adults with a severe fear of falling are more likely to suffer from MCR. Studies have shown that decreased balance function, slow gait speed, and negative emotions can induce and aggravate the fear of falling (40). The cognitive motor risk composite is characterized by slow gait and subjective cognitive decline (41), which can lead to negative emotions (34). Therefore, older adults with cognitive motor risk syndrome are more likely to experience a fear of falling.

Older adults with chronic pain are more likely to suffer from MCR. Some studies (42) have shown that the severity of pain is closely related to the increased risk of MCR. This may be because chronic pain accelerates cognitive decline and slows gait speed in older adults (43). In addition, those living with children are more likely to suffer from MCR. No studies have addressed this point, but it may be related to overprotection. Older adults cared for by their children may experience decreased activities of daily living, memory and executive function.

### Guidance for future research and practice

Dementia burdens families, healthcare systems and societies (44). Currently, effective treatments for dementia are still lacking. Therefore, attention to predementia syndromes and their modifiable risk factors is critical. MCR is a predementia syndrome similar to mild cognitive impairment (MCI) (45), so future research should focus on MCR interventions. In rural areas, a support system can be established to increase social support for older adults living alone, providing them security and a sense of belonging. Future interventions could include physical activity (30), cognitive behavioral therapy (21), an anti-inflammatory diet (46), pain management (42), and medication management (24) in primary healthcare settings. These interventions would help strengthen their constitutions, reduce chronic pain, and improve mood and cognitive abilities. Professionals have attempted brain stimulation interventions (36), dual-task training (37), music interventions (47) and other approaches to improve memory problems and executive disorders in older adults. These interventions are beneficial for reducing the incidence of MCR and dementia. Few studies use professional instruments to measure related variables, and the data lacks objectivity. Future studies should focus on using professional tools to measure these variables, thereby improving the accuracy of the findings.

### Strengths and limitations

This study has several strengths. First, the large sample size improves the accuracy and reliability of the data analysis results. Second, few studies have investigated the influence of MCR in rural older populations in China. This study focuses on the rural older population, providing an in-depth understanding of this group’s incidence and related influencing factors of MCR. It should be noted that this study also has the following limitations. First, due to the cross-sectional design and lack of long-term follow-up, true causality cannot be determined. Second, the single-center nature of the study limits the generalisability of the findings. Third, the assessment tools are predominantly questionnaire scales, which introduces a degree of subjectivity. Fourth, Convenience sampling was used in this study, and there is a possibility of selection bias.

## Conclusion

This study revealed that the prevalence of MCR among Chinese rural older adults was 11.66%. Fear of falling, pain, age, depression, social support, living conditions, types of medication, vision loss, and chronic diseases were the influencing factors of MCR. Therefore, personalized interventions should be developed for rural older adults to improve their motor ability and cognitive function according to the modifiable influencing factors identified in this study. This would be beneficial for preventing the occurrence of dementia and improving quality of life.

## Data Availability

The original contributions presented in the study are included in the article/supplementary material, further inquiries can be directed to the corresponding authors.
